# Extremely Large Non-equilibrium Tunnel Magnetoresistance Ratio in CoRhMnGe Based Magnetic Tunnel Junction by Interface Modification

**DOI:** 10.3389/fchem.2019.00550

**Published:** 2019-08-27

**Authors:** Yu Feng, Zhenxiang Cheng, Xiaotian Wang

**Affiliations:** ^1^Laboratory for Quantum Design of Functional Materials, School of Physics and Electronic Engineering, Jiangsu Normal University, Xuzhou, China; ^2^Institute for Superconducting and Electronic Materials, University of Wollongong, Wollongong, NSW, Australia; ^3^School of Physical Science and Technology, Southwest University, Chongqing, China

**Keywords:** magnetic tunnel junction, heusler alloys, interface modification, tunnel magnetoresistance, nonequilibrium green's function

## Abstract

Equiatomic quaternary Heusler compounds (EQHCs) generally have the advantages of high Curie temperature, large spin polarization and long spin diffusion length, and they are regarded as one of the most promising candidates for spintronics devices. Herein, we report a theoretical investigation on an EQHC CoRhMnGe based magnetic tunnel junction (MTJ) with (i) MnGe-terminated interface and (ii) modified pure Mn terminated interface, i.e., MnMn-terminated interface. By employing first principle calculations combined with non-equilibrium Green's function, the local density of states (LDOS), transmission coefficient, spin-polarized current, tunnel magnetoresistance (TMR) ratio and spin injection efficiency (SIE) as a function of bias voltage are studied. It reveals that when the MTJ under equilibrium state, TMR ratio of MnGe-terminated structure is as high as 3,438%. When the MTJ is modified to MnMn-terminated interface, TMR ratio at equilibrium is enhanced to 2 × 10^5^%, and spin filtering effects are also strengthened. When bias voltage is applied to the MTJ, the TMR ratio of the MnGe-terminated structure suffers a dramatic loss. While the modified MnMn-terminated structure could preserve a large TMR value of 1 × 10^5^%, even bias voltage rises up to 0.1 V, showing a robust bias endurance. These excellent spin transport properties make the CoRhMnGe a promising candidate material for spintronics devices.

## Introduction

Spintronics, with the manipulation of electron spin as information carrier, has the advantages of higher circuit integration density, fast operation and less energy consumption (Wolf et al., 2001; Žutić et al., 2004; Li and Yang, 2016). As one of the most important spintronic devices, magnetic tunnel junction (MTJ), consisting of two ferromagnetic (FM) electrodes separated by a thin non-magnetic (NM) semiconductor layer, has attracted great attention (Li et al., 2014; Iqbal et al., 2016; Wang et al., 2016a). Magnetic field sensors utilizing tunnel magnetoresistance (TMR) effects possess a better signal-to-noise ratio, and magnetic random access memory (MRAM) based on TMR effects has higher data rates and could further enhance the record density and minimize device dimension (Butler et al., 2001; Mao et al., 2006). However, the reliability of a MTJ device is seriously affected by high bias current owing to the current-driven instabilities and electromigration. Therefore, high TMR ratios, necessary to achieve sufficient output signal under moderate current densities, is a crucial indicator for a high performance MTJ device. According to Valet-Fert model, i.e., two-current model (Valet and Fert, 1993), TMR effect is proportional to spin asymmetry coefficients for FM layer (β) and FM/NM interface (γ). On the one hand, β is determined by the electronic band structure of FM layers, thus half-metallic ferromagnet (HMF) which owns complete spin polarization has been regarded as one of the most promising materials to work as electrode (Wen et al., 2014; Feng et al., 2019). Among various HMFs, Heusler compounds received considerable interest mainly because they have a high Curie temperature and small lattice mismatching between Heusler compound and conventional semiconductor (Graf et al., 2011; Skaftouros et al., 2013; Nayak et al., 2015; Zhang et al., 2015; Bainsla and Suresh, 2016; Sahoo et al., 2016; Wang et al., 2016b, 2017; Han et al., 2017; Jamer et al., 2017; Siakeng et al., 2018). Extensive attentions have been paid to MTJ employing the Heusler compound as a spin injector such as Co_2_FeAl/MgO (Scheike et al., 2014; Wen et al., 2014), Co_2_(Mn, Fe)Si/MgO (Moges et al., 2016), Co_2_MnSi/MgO (Yamamoto et al., 2010; Kozina et al., 2014), Fe_3_Si/MgO (Tao et al., 2014) and CoFeMnSi/MgO (Bainsla et al., 2018). More recently, the equiatomic quaternary Heusler compound CoRhMnGe has been successfully synthesized (Rani et al., 2017). It demonstrates half metallicity with high Curie temperature of ~760 K, revealing great potential for spintronic devices. On the other hand, the coefficient γ is governed by the electronic structure of FM/NM interface, because spin-dependent asymmetric scattering at FM/NM interface may dominate electron tunneling (Sakuraba et al., 2010). One way to enhance the TMR effect is to engineer the interface electronic structure by interface modification. The study on the Co_2_MnSi (001) surface revealed that surface half metallicity in all natural terminations is destroyed due to the appearance of surface states; however, when the surface was modified to pure Mn termination (i.e., MnMn-termination), it maintains the half metallicity due to the strong surface-subsurface coupling (Hashemifar et al., 2005). Other studies on Co_2_MnX (X = Si, Ge, Sn) (Wu et al., 2012), Co_2_MnGe_0.5_Ga_0.5_ (Wu et al., 2011), and CoFeMnSi/GaAs (Feng et al., 2015) heterojunction also confirmed that modified surface/interface could preserve 100% spin polarization.

In this work, we build CoRhMnGe/MgO/CoRhMnGe MTJ with (i) MnGe-terminated interface and (ii) modified pure Mn terminated interface (i.e., MnMn-terminated interface), and employ the non-equilibrium Green's function in combination with first principles calculations to study the non-equilibrium spin injection and spin-polarized quantum transport properties. Our results show that CoRhMnGe/MgO/CoRhMnGe MTJ possess extremely large TMR value, and such value could be further enhanced when the interface of the MTJ is modified to Mn-rich termination.

### Calculation Method

**Figure 1:**
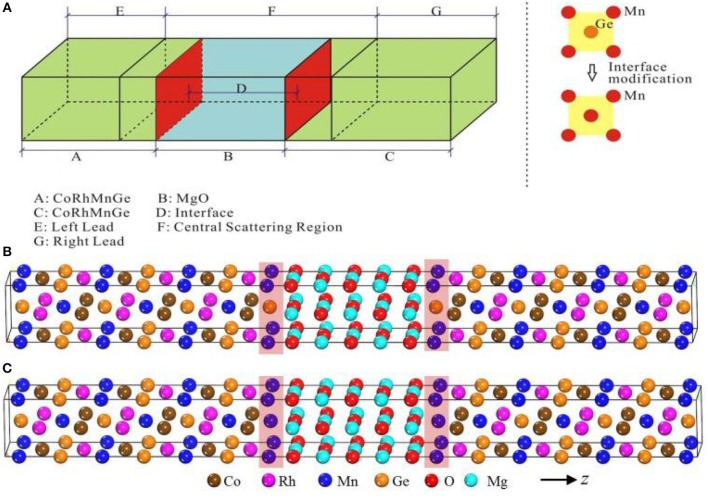
**(A)** Schematic sketch of the CoRhMnGe/MgO/CoRhMnGe MTJ. **(B)** MnMn-terminated structure. **(C)** MnGe-terminated structure. The *z*-axis is the transport direction.

In Figure 1, a two-probe MTJ device model consisting of two semi-infinite CoRhMnGe electrodes sandwiching 5 MgO layers is built, and the model is divided into left electrode, central scattering region and right electrode. The modified pure Mn terminated interface (i.e., MnMn-terminated interface) is obtained by substituting the interface Ge by Mn in MnGe-terminated interface. The in-plane lattice constant of the junction is fixed at 4.21 Å which is the value of MgO. Besides, the $1/\sqrt{2}$ of experimental lattice constant of CoRhMnGe (5.89 Å) is 4.16 Å, and the lattice mismatch between CoRhMnGe and MgO is about 1%. The interface relaxation is firstly performed by using VASP package based on density functional theory (DFT) (Blöchl, 1996; Perdew et al., 1996). The self-consistent field (SCF) convergence criterion of 10^−6^ eV and mesh of 9 × 9 × 1 Monkhorst-Pack k-points in Brillouin zone are applied, and the cut-off energy is set as 500 eV. The optimized distance between the CoRhMnGe layer with MnGe-terminated interface and MgO layer is found to be 2.42 Å, while that between the CoRhMnGe layer with MnMn-terminated interface and MgO layer is found to be 2.02 Å. The spin-dependent transport properties calculations are based on a state-of-the-art technique where DFT is combined with the Keldysh non-equilibrium Green's function (NEGF) theory, as implemented in Nanodcal package (Taylor et al., 2001; Waldron et al., 2006). In our work, the MTJ device model is periodic along the *x*- and *y* axes, while the transport direction is along the *z* axis. In our calculations of transport properties, the number of Monkhorst-Pack k-space grids of left and right electrode is 10 × 10 × 100, and that of central scattering region is 10 × 10 × 1, and the self-consistent calculations are limited to 10^−5^ Hatree tolerance.

## Results and Discussion

**Figure 2:**
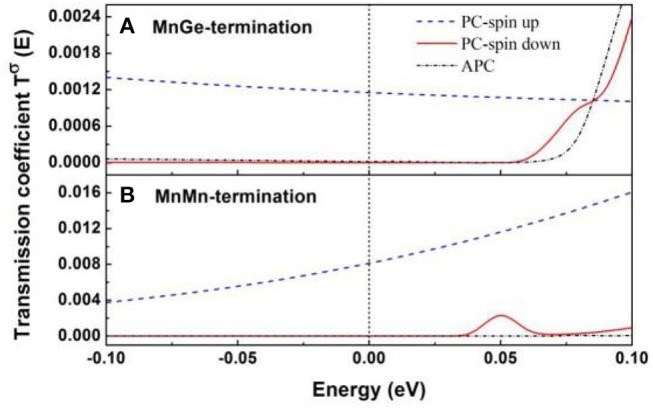
Zero bias transmission coefficient versus electron energy in PC and APC of the CoRhMnGe/MgO/CoRhMnGe MTJ with **(A)** MnGe-termination and **(B)** MnMn-termination.

In Figure 1, the two-probe MTJ device model, consisting of two semi- MnGe-terminated structures and MnMn-terminated structures, are based on converged SCF calculations of the center scattering region and two electrodes. The energy- and spin-dependent transmission coefficient *T*^σ^(*E*) can be calculated by $T^{\sigma }\left(E\right)=Tr\left[\Gamma_{L}G^{R}\Gamma_{R}G^{A}\right]$, where σ is spin direction (up or down); Γ_L_ and Γ_R_ are the coupling matrix of the left electrode and right electrode, respectively; and G^R^ and G^A^ are the retarded and advanced Green's function of the central region, respectively. The transmission coefficient vs. electron energy for MnGe-terminated and MnMn-terminated structures at equilibrium are calculated and displayed in Figure 2. (i) When two magnetic electrodes are in antiparallel magnetization configuration (APC), due to the reason that these two structures are mirror-symmetrical with respect to the middle of the scattering region, the transmission coefficient curve in spin up channel completely coincide with that in spin down channel. Hence, there is only one transmission coefficient curve in APC (dashed-dotted black line). (ii) When two magnetic electrodes are in parallel magnetization configuration (PC), the transmission coefficient curve in spin up channel is totally different from that in spin down channel. Clearly, for both MnGe-terminated and MnMn-terminated structures, transmission coefficient at Fermi level in spin up channel ($T_{PC}^{up}\left(E_{f}\right)$) is much higher than that in spin down channel ($T_{PC}^{down}\left(E_{f}\right)$). Therefore, the total transmission coefficient at Fermi level in PC mainly comes from the contribution of the spin up electrons.

Physically, tunnel magenetoresistance ratio (TMR) indicates the sensitivity of a MTJ device with respect to the magnetic configuration, and is regarded as one of the most important parameters to characterize performance of a MTJ device. The TMR ratio at zero bias in our work is calculated by the following definition:

$$\begin{array}{l} TMR=\left|\frac{T_{pc}\left(E_{f}\right)-T_{APC}\left(E_{f}\right)}{\min\left(T_{pc}\left(E_{f}\right),\;T_{APC}\left(E_{f}\right)\right)}\right| \end{array}$$

where *T*_*PC*_(*E*_*f*_) and *T*_*APC*_(*E*_*f*_) denote total transmission coefficients at Fermi level in PC and APC, and they are obtained by summing up the contributions of the spin up and spin down channel, thus $T_{PC}\left(E_{f}\right)=T_{PC}^{up}\left(E_{f}\right)+T_{PC}^{down}\left(E_{f}\right)$ and $T_{APC}\left(E_{f}\right)=T_{APC}^{up}\left(E_{f}\right)+T_{APC}^{down}\left(E_{f}\right)$; min(*T*_*pc*_(*E*_*f*_), *T*_*APC*_(*E*_*f*_)) is the smaller one of *T*_*PC*_(*E*_*f*_) and *T*_*APC*_(*E*_*f*_). For MnGe-terminated structures, the calculated $T_{PC}^{up}\left(E_{f}\right)$ and $T_{PC}^{down}\left(E_{f}\right)$ are 0.0012 and 0, respectively; and $T_{APC}^{up}\left(E_{f}\right)=T_{APC}^{down}\left(E_{f}\right)=0.1696\times 10^{-4}$. The TMR ratio of CoRhMnGe/MgO/CoRhMnGe MTJ with MnGe-termination at equilibrium is calculated to be 3,438%. Besides, for MnMn-terminated structures, the calculated $T_{PC}^{up}\left(E_{f}\right)$ and $T_{PC}^{down}\left(E_{f}\right)$ are 0.0081 and 0, respectively; and $T_{APC}^{up}\left(E_{f}\right)=T_{APC}^{down}\left(E_{f}\right)=0.2054\times 10^{-5}$. The calculated TMR ratio of CoRhMnGe/MgO/CoRhMnGe MTJ with MnMn-termination at equilibrium reaches up to about 2 × 10^5^%. It indicates that TMR ratio of CoRhMnGe/MgO/CoRhMnGe MTJ at equilibrium can be greatly enhanced by modifying the interface to MnMn-termination.

**Figure 3:**
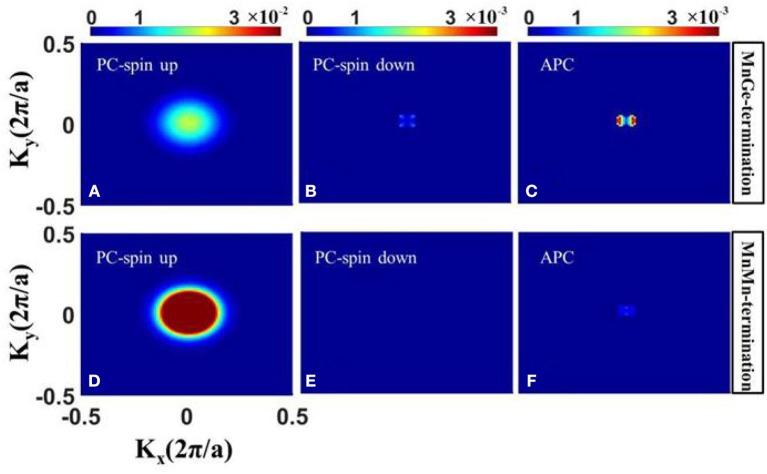
The *k*_//_-resolved transmission coefficients at the Fermi level *E* = *E*_*f*_ of CoRhMnGe/MgO/CoRhMnGe MTJ with MnGe-termination **(A–C)** and MnMn-termination **(D–F)**.

Furthermore, spin dependent *k*_//_-resolved transmission in 2-D BZ at Fermi level is also calculated. Figure 3 displays the contour plots of the *k*_//_dependence of the spin up and spin down transmission coefficient at E = E_*f*_ in PC and APC. (i) In APC, due to the reason we have mentioned above in the transmission curve discussion, the contour plot of transmission coefficient in spin up channel is exactly the same as that in spin down channel, and there is only one transmission contour plot in APC (see Figures 3C,F). There are little *hot spots* (Wunnicke et al., 2002) that exist in APC, indicating that the transmission of spin polarized electron has been blocked. (ii) In PC, the dense of *hot spots* in spin up channel (see Figures 3A,D) are much larger than that in spin down channel (see Figures 3B,E), indicating that in PC the spin up electrons have higher transport ability than spin down electrons, which is in agreement with transmission curves shown in Figure 2. Moreover, comparing transmission contour plot of these two structures, it can be seen that in PC the *hot spots* in spin up channel of the MnMn-terminated structure (see Figure 3D) are much hotter than that of the MnGe-terminated structure (see Figure 3A). Besides, in APC the dense of *hot spots* of the MnMn-terminated structure (see Figure 3F) are much weaker than that of the MnGe-terminated structure (see Figure 3C). Therefore, by modifying the interface of CoRhMnGe/MgO/CoRhMnGe MTJ to MnMn-termination, the transport ability of spin up electron in PC is much improved and the spin polarized electron in APC suffers a stronger suppression, leading to an obvious enhancement in TMR ratio.

**Figure 4:**
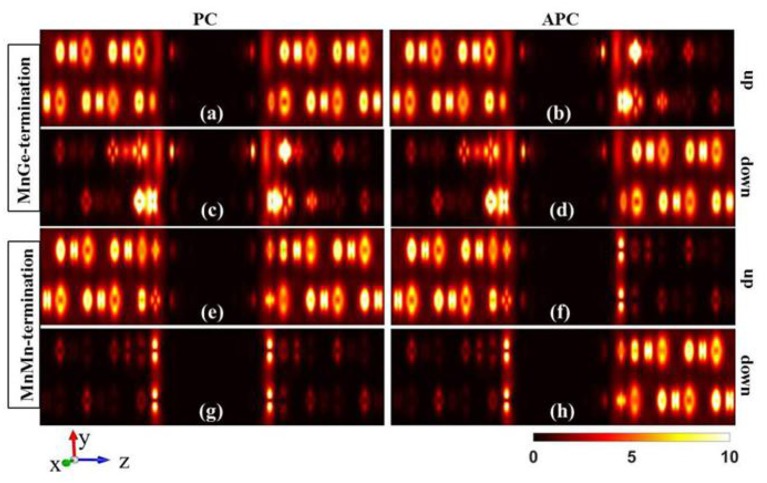
The local density of states (LDOS) at the Fermi level of CoRhMnGe/MgO/CoRhMnGe MTJ with MnGe-termination **(a–d)** and MnMn-termination **(e–h)**. LDOSs in PC and in APC are listed in the left column and right column, respectively. LDOSs in spin up channels are listed in **(a,b,e,f)**. LDOSs in spin down channels are listed in **(c,d,g,h)**.

The excellent transport properties in MnMn-terminated structures could be intuitively understood by analyzing the local density of states (LDOS). The LDOS of the MnGe-terminated structure and MnMn-terminated structure at equilibrium state are calculated and summed along the transport direction (*z*-axis direction). As shown in Figure 4, a state gap resulting from semiconductor MgO exists in the two investigated structures, revealing the tunneling transport mechanism. (i) In PC, it can be seen from Figures 4a,e that both left and right electrodes possess large density of states in spin up channel and it reveals that spin up channel is free, and abundant number of spin up electrons can tunnel through from left electrode into right electrode. Spin up state is the majority spin state. Besides, in spin down channel, there are few densities of states in two electrodes (see Figures 4c,g), indicating that spin down channel is suppressed and spin down electrons can hardly transmit via the magnetic electrode by tunneling through the MgO barrier, and spin down state is the minority spin state. Therefore, when these two investigated structures are under PC state, polarized current dominated by spin up electrons can tunnel through the MgO barrier, and they are in a low resistance state. (ii) In APC, we can see from Figures 4b,f that in spin up channel only left electrode possesses large density of states, while right electrode has few densities of states. It reveals that although left electrode can generate spin up electrons, there are few states in right electrode can accommodate these electrons, and spin up electrons cannot flow into right electrode. In opposition, in spin down channel (see Figures 4d,h) large density of states are concentrated on right electrode, while few density of states exist in left electrode. It indicates that although right electrode can accept lots of electrons, the left electrode can barely offer spin down electrons. Therefore, when these two structures are under APC state, both spin up and spin down channels are blocked, and spin polarized current cannot be detected, leading to a high resistance state. Moreover, comparing Figure 4c with Figure 4g, we can see that under PC state the spin down density of states of the MnMn-terminated structure is much weaker than that of the MnGe-terminated structure, revealing that the purity of spin polarized current of the MnMn-terminated structure is higher than that of the MnGe-terminated structure. Therefore, spin filtering effect in CoRhMnGe/Mg/CoRhMnGe MTJ get enhanced by modifying the interface to MnMn-termination. In addition, when these two structures are under APC state, the spin up density of states in right electrode of the MnMn-terminated structure is much weaker than that of the MnGe-terminated structure (see Figures 4b,f), and the same situation can also be observed in spin down density of states in left electrode (see Figures 4d,h). It indicates that spin polarized current of MnMn-terminated structure suffered more intense suppression under APC state.

**Figure 5:**
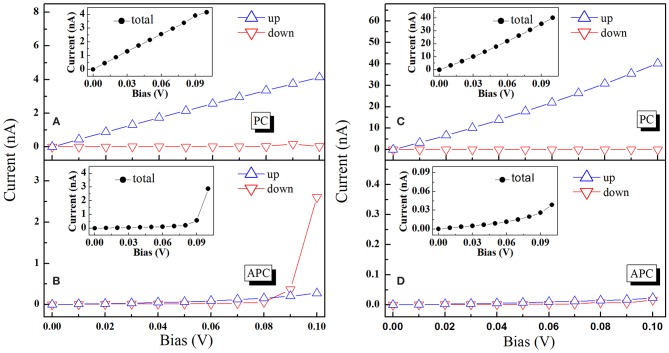
**(A,B)** are for the MnGe-terminated structure. **(A)** I-V curves in PC and **(B)** I-V curves in APC. **(C,D)** are for the MnMn-terminated structure. **(C)** I-V curves in PC and **(D)** I-V curves in APC.

Now we turn our discussion to spin transport properties at non-equilibrium. The spin polarized currents of MnGe-terminated and MnMn-terminated structures as the function of bias voltage are calculated and presented in Figure 5. The total spin polarized currents in PC ($I_{PC}^{tot}$) and APC ($I_{APC}^{tot}$) are represented by black spots in the insets. The blue triangle and red triangle are spin up current (*I*^*up*^) and spin down current (*I*^*down*^), respectively. Here, *I*^*tot*^ = *I*^*up*^ + *I*^*down*^. (i) In PC, spin up current increases linearly with bias voltage up to 0.1 V whilst spin down current always remains minor value and is unresponsive to the increase of bias voltage. Besides, the total currents of these two structures also linearly increase with the increase of bias. (ii) In APC, spin up, spin down and total currents of MnMn-terminated structure always hold minor values when bias ranges from 0 to 0.1 V, indicating that the spin polarized currents of MnMn-terminated structures are significantly inhibited in APC. For MnGe-terminated structures, spin up and spin down currents almost keep the minor values unchanged when bias increases from 0 to 0.09 V, while when bias exceeds the 0.09 V and increases to 0.1 V, spin down current rapidly increases to a very high value of 2.6 nA (see red triangle in Figure 5B). Moreover, comparing Figure 2A with Figure 2B, we can see that in PC the spin up current intensity of the MnMn-terminated structure is about 10 times larger than that of the MnGe-terminated structure.

**Figure 6:**
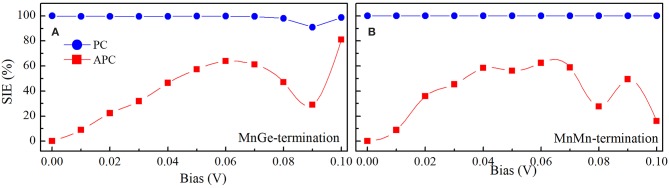
Spin injection efficient (SIE) as a function of bias in PC and APC for **(A)** MnGe-terminated structure and **(B)** MnMn-terminated structure.

The spin injection efficiency (SIE) is an important parameter that can reveal the degree of the spin polarization in the transport current, and it can be expressed as $SIE=\left|\frac{I^{up}-I^{down}}{I^{up}+I^{down}}\right|\times 100\%$. The SIE in PC and APC are calculated and displayed in Figure 6. (i) In PC, SIE of these two structures always maintains a high value of nearly 100% when bias incessantly rises up to 0.1 V, and this can be explained by the reason that in PC the spin up current is much larger than the spin down current which could be observed from Figures 5A,C. Hence, these two structures could obtain nearly 100% spin polarized current in PC configuration. In APC, SIE of the MnGe-terminated structure nearly linearly increases to 64% with bias up to 0.06 V, and then it drops to 29% when bias increases to 0.09 V; while it dramatically reaches up to 81% when bias up to 0.1 V. For the MnMn-terminated structure, SIE in APC firstly increases monotonously to 58% when bias increases to 0.04 V, and then it maintains a relative stable value of about 60% when bias ranges from 0.04 to 0.07 V, and it begins to deteriorate gradually to a poor value of 16% at 0.1 V bias.

**Figure 7:**
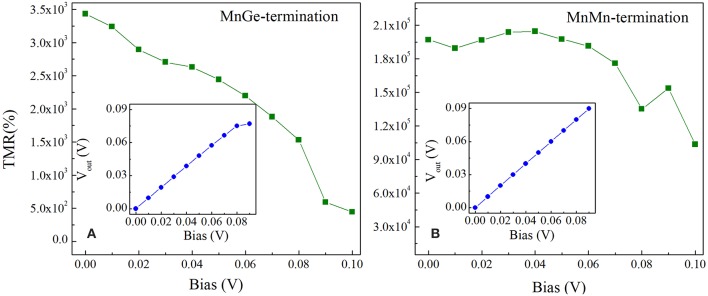
TMR ratio and output voltage V_out_ (inset) as a function of bias for **(A)** MnGe-terminated structure and **(B)** MnMn-terminated structure.

In our work, TMR ratio under finite bias is defined as $TMR=\left|\frac{I_{PC}^{tot}-I_{APC}^{tot}}{\min\left(\overset{tot}{\underset{PC}{I}},I_{APC}^{tot}\right)}\right|\times 100\%$. Figure 7 exhibits the TMR ratio vs. bias voltage for the two investigated structures under non-equilibrium. The TMR ratio of MnGe-terminated descends rapidly with increasing of bias, and eventually drops to about 500% at a bias of 0.1 V; its TMR ratio loss rate (η = (*TMR*_max_−*TMR*_min_)/*TMR*_max_) exceeds 80%, where TMR_max_ (or TMR_min_) is the maximal (or minimal) TMR ratio within the range of bias voltage variation. However, the maximal TMR ratio of MnMn-terminated structure reaches up to about 2 × 10^5^%, and it maintains such large value when bias ranges from 0 to 0.06 V. More importantly, when bias increases to 0.06 V, the TMR ratio loss rate of MnMn-terminated structure is only about 6%, and its TMR ratio is still higher than 1 × 10^5^% even for bias up to 0.1 V. It indicates that TMR ratio under bias voltage is greatly enhanced by modifying the interface of CoRhMnGe/MgO/CoRhMnGe MTJ to MnMn-termination, and TMR ratio of such modified structure is much less affected by the bias voltage, showing a robust bias endurance. Besides, in order to characterize the magnitude of the output signal modulation (Tiusan et al., 2006), the output voltage V_out_ is calculated by $V_{out}=V_{b}\left(I_{PC}^{tot}-I_{APC}^{tot}\right)/I_{PC}^{tot}$, where V_b_ is the applied voltage. In the inset of Figure 7A, the V_out_ of MnGe-terminated structure increases linearly with bias voltage increases to 0.08 V and then drops at 0.09 V bias owing to the severe suppression of TMR ratio by bias. However, V_out_ of MnMn-terminated structure increases in a strict linearly manner when bias ranges from 0 to 0.1 V (see the inset of Figure 7B).

**Figure 8:**
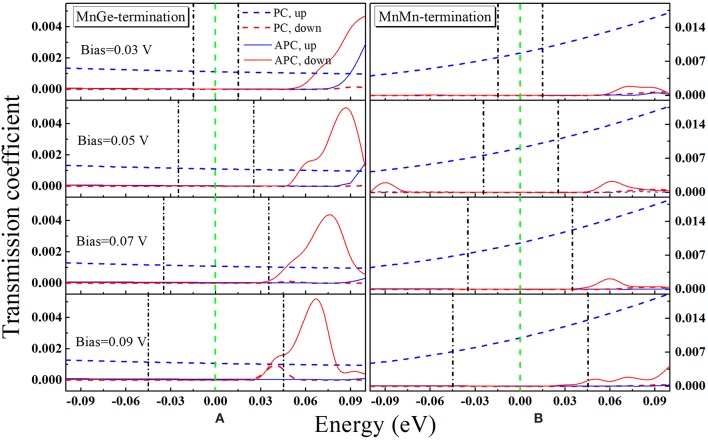
Transmission coefficient vs. electron energy for **(A)** MnGe-terminated structure and **(B)** MnMn-terminated structure at different bias. The bias window in each panel is between the two green vertical dash lines. Note that in order to show the trend of the transmission coefficient curve of MnMn-terminated structure in APC more clearly, these curves are zoomed in 10 times, see blue and red solid lines in column **(B)**.

In order to further understand the TMR behaviors of MnGe-terminated and MnMn-terminated structures at non-equilibrium state, transmission coefficients as a function of electron energy at a finite bias for these two structures are calculated and presented in Figure 8. Bias window is set to the center region between two black dashed-dotted lines, and current is obtained by integral of transmission coefficients over bias window−*V*/2 ≤ *E* ≤ +*V*/2, i.e., $I\tilde{\;}\int_{-V/2}^{+V/2}T\left(E,V\right)dE$. Note that to show the trend of the transmission coefficient curve of the MnMn-terminated structure in APC more clearly, these curves are zoomed in 10 times (see blue and red solid lines in column (b) in Figure 8). In APC, due to the reason that the geometric symmetry is broken by the bias, the transmission curve of the spin up channel is no longer the same as that of the spin down channel. (i) For the MnGe-terminated structure, in PC the transmission curves within the bias window have not changed much while, in APC, the spin down transmission curves own a peak around E = 0.1 eV at bias of 0.03 V, and this peak gradually moves toward the bias window with the increasing of bias. Besides, in APC more and more spin down transmission curves enter the bias window and spin down transmission coefficient in APC gets enhanced. Therefore, TMR ratio of the MnGe-terminated structure suffers a rapid decrease with the increasing of bias. (ii) For the MnMn-terminated structure, because in PC the value of spin up transmission curves are much larger than the MnGe-terminated structure, spin up current of the former structure is much higher than that of the latter structure, which can be observed in Figures 5A,C. In APC, there is a peak around E = 0.08 eV in spin down channel, and such a peak shifts to enter the bias window with the increasing of bias to make more and more contribution to transmission coefficient in APC. Although transmission coefficients in APC are strengthened, they are still much lower than that in PC, therefore an ultra-high TMR ratio can be maintained under large bias in the MnMn-terminated structure.

## Conclusions

In conclusion, in order to explore the potential applications of CoRhMnGe in spintronics device, we performed non-equilibrium Green's function combined with the first principle calculations to investigate the spin transport properties of CoRhMnGe/MgO/CoRhMnGe MTJ. The local density of states (LDOS), transmission coefficient, spin-polarized current, tunnel magnetoresistance (TMR) ratio and spin injection efficiency (SIE) as a function of bias voltage are studied. Our calculation reveals that when CoRhMnGe/MgO/CoRhMnGe MTJ under equilibrium state, the TMR ratio of MnGe-terminated structure is as high as 3,438%, while that of modified MnMn-terminated interface can reach up to 2 × 10^5^%, and spin filtering effects also get strengthened by interface modification. When bias voltage is applied to the MTJ, the TMR ratio of the MnGe-terminated structure suffers a dramatic loss. While the modified MnMn-terminated structure could preserve a large TMR value even if bias up to 0.1 V is applied, showing a robust bias endurance. These excellence spin transport properties of CoRhMnGe based magnetic tunnel junctions with modified interface atoms make it a promising candidate material for future spintronics devices.

## Data Availability

The raw data supporting the conclusions of this manuscript will be made available by the authors, without undue reservation, to any qualified researcher.

## Author Contributions

All authors listed have made a substantial, direct and intellectual contribution to the work, and approved it for publication.

### Conflict of Interest Statement

The authors declare that the research was conducted in the absence of any commercial or financial relationships that could be construed as a potential conflict of interest.
